# Visualization and Analysis of Microtubule Dynamics Using Dual Color-Coded Display of Plus-End Labels

**DOI:** 10.1371/journal.pone.0050421

**Published:** 2012-11-30

**Authors:** Amy K. Garrison, Mahalakshmi Shanmugam, Haiwen Connie Leung, Caihong Xia, Zheng Wang, Le Ma

**Affiliations:** 1 Zilkha Neurogenetic Institute, Keck School of Medicine, University of Southern California, Los Angeles, California, United States of America; 2 Program in Genetic, Molecular and Cellular Biology, Keck School of Medicine, University of Southern California, Los Angeles, California, United States of America; 3 Department of Cell and Neurobiology, Keck School of Medicine, University of Southern California, Los Angeles, California, United States of America; Vanderbilt University Medical Center, United States of America

## Abstract

Investigating spatial and temporal control of microtubule dynamics in live cells is critical to understanding cell morphogenesis in development and disease. Tracking fluorescently labeled plus-end-tracking proteins over time has become a widely used method to study microtubule assembly. Here, we report a complementary approach that uses only two images of these labels to visualize and analyze microtubule dynamics at any given time. Using a simple color-coding scheme, labeled plus-ends from two sequential images are pseudocolored with different colors and then merged to display color-coded ends. Based on object recognition algorithms, these colored ends can be identified and segregated into dynamic groups corresponding to four events, including growth, rescue, catastrophe, and pause. Further analysis yields not only their spatial distribution throughout the cell but also provides measurements such as growth rate and direction for each labeled end. We have validated the method by comparing our results with ground-truth data derived from manual analysis as well as with data obtained using the tracking method. In addition, we have confirmed color-coded representation of different dynamic events by analyzing their history and fate. Finally, we have demonstrated the use of the method to investigate microtubule assembly in cells and provided guidance in selecting optimal image acquisition conditions. Thus, this simple computer vision method offers a unique and quantitative approach to study spatial regulation of microtubule dynamics in cells.

## Introduction

**Figure 1:**
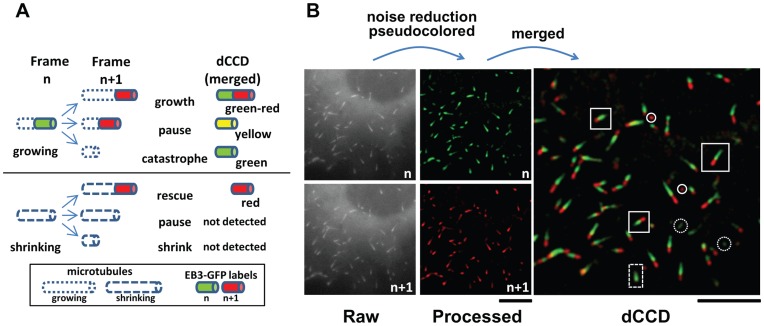
General strategy of the dCCD method. A. Illustrations of microtubule behaviors underlying the rational of the dCCD method. Growing microtubules (labeled by EB3-GFP at plus ends) can grow, pause, or undergo catastrophe, while shrinking microtubules (lacking EB3-GFP) can be rescued, pause, or continue to shrink. When EB3-GFP labels from two sequential images are pseudocolored (green in the *n*th frame and red in the *n+1*th frame), their relative position in the merged dCCD images generates different color combinations or codes representing four of these dynamic events. Green-reds represent growing ends, as EB3-GFP labels advance along newly-added tubulin at the tip of polymerizing microtubules. Red marks ends which regain EB3-GFP labels in the *n+1*th frame, and indicates rescue from shrinkage to growth. Green reveals ends undergoing catastrophe and losing EB3-GFP labels in the *n+1*th frame, while yellow labels pausing ends with EB3-GFP present in the same location of both frames. **B.** Construction of a dCCD image. Two raw fluorescent images acquired at 5 sec intervals from a COS cell expressing EB3-GFP were processed to remove background and then pseudocolored, with the first image (*n*th) in green and the following image (*n+1*th) in red. They were merged to generate a dCCD image. Four types of color-coded ends can be readily seen (green-red: solid rectangle, red: solid circle, green: dashed circle, and yellow: dashed rectangle). Scale bars: 5 µm.

Microtubule assembly in cells is characterized by stochastic conversion between phases of growth and shrinkage at the plus ends, a property known as dynamic instability [1]. These two phases and the transitions between them, including rescue, catastrophe and pause (Figure 1A), are targets for regulation in many cellular processes, such as mitosis, cell polarization, directed movement, and nerve guidance [1], [2], [3], [4]. Knowledge of their spatial distribution is thus crucial to understanding the function and regulation of microtubule assembly in these complex processes.

Fluorescently labeled tubulin proteins were the first probes developed to image microtubule dynamics in live cells [5], [6], [7], but the high density of microtubules limits analysis of dynamics to the cell periphery or demands special tools, such as fluorescent speckle microscopy [8], [9]. Recently, fluorescently tagged plus-end-tracking proteins (+TIPs) have provided a new probe to overcome this limitation [10]. These proteins preferentially associate with growing plus-ends with fast on- and off-rates, allowing the visualization of microtubule plus-ends in the entire cell [10], [11]. Both manual and automated methods have been developed to measure microtubule dynamics by tracking the movement of comet-like +TIP labels in sequential image stacks [12], [13], [14], [15]. However, the current tracking algorithms require frequent fluorescence imaging that is not always feasible in rapidly moving cells and is sometimes damaging to delicate subcellular structures such as growth cones.

To provide a rapid way to assess microtubule plus-end dynamics, we developed a dual color-coded display (dCCD) method to extract dynamic information from only two images at any given time. Based on the temporal and spatial relationship of a +TIP label (such as EB3-GFP) at microtubule ends in two sequential images, this method generates color codes that represent four dynamic events, including growth, rescue, catastrophe and pause. Object recognition algorithms can then be used to identify and segregate these color-coded ends, and to obtain measurements of their distribution as well as growth parameters throughout the cell. We present data to validate color representation of different dynamic events and show that measurements are comparable to those obtained with the tracking method. Thus, the dCCD method offers a novel approach to studying microtubule dynamics in space.

## Materials and Methods

### EB3-GFP Imaging in Cultured Cells

COS cells were grown on coverslips in DMEM supplemented with 10% fetal bovine serum. They were transfected with plasmid DNA expressing EB3-GFP [16] (a gift from Neils Galjart) using Fugene-6 (Roche). Cells on coverslips were moved to a custom made culture chamber 16–20 hrs after transfection and grown at 32°C on an inverted microscope (Axiovert 200, Zeiss). Culture medium was replaced with fresh medium supplemented with 10 mM HEPES (pH 7.4). GFP fluorescence was excited by light emitted from a 100-W mercury lamp and attenuated with a neural density filter (10–25%), and imaged with a 63× apochromatic objective (N.A. = 1.4) and a 2.5× optovar. Time-lapse images were collected by an EMCCD camera (Cascade II, Photometrics) with 300–500 ms exposure time and 5 sec interval using the Metamorph software (Molecular Devices).

### dCCD Analysis

Imaging processing, dCCD image assembly, and end identification, segregation and analysis were all done with computer programs written in MATLAB (Mathworks) following the work flow in Figure S1. Briefly, raw fluorescent images (16 bit, Figure 1B and S2A) were processed to eliminate background noise using the difference of two Gaussian filters (σ_Low_ = 1, σ_high_ = 4). A Wiener filter can be used to further reduce noise here. The resulting processed images were converted to 8-bit images using a user-defined scaling factor (default = 2). These processed images were then pseudocolored in separate green (*n*th frame) or red (*n+1*th frame) color and merged to generate the dCCD images (Figure 1B and S2B).

**Figure 2:**
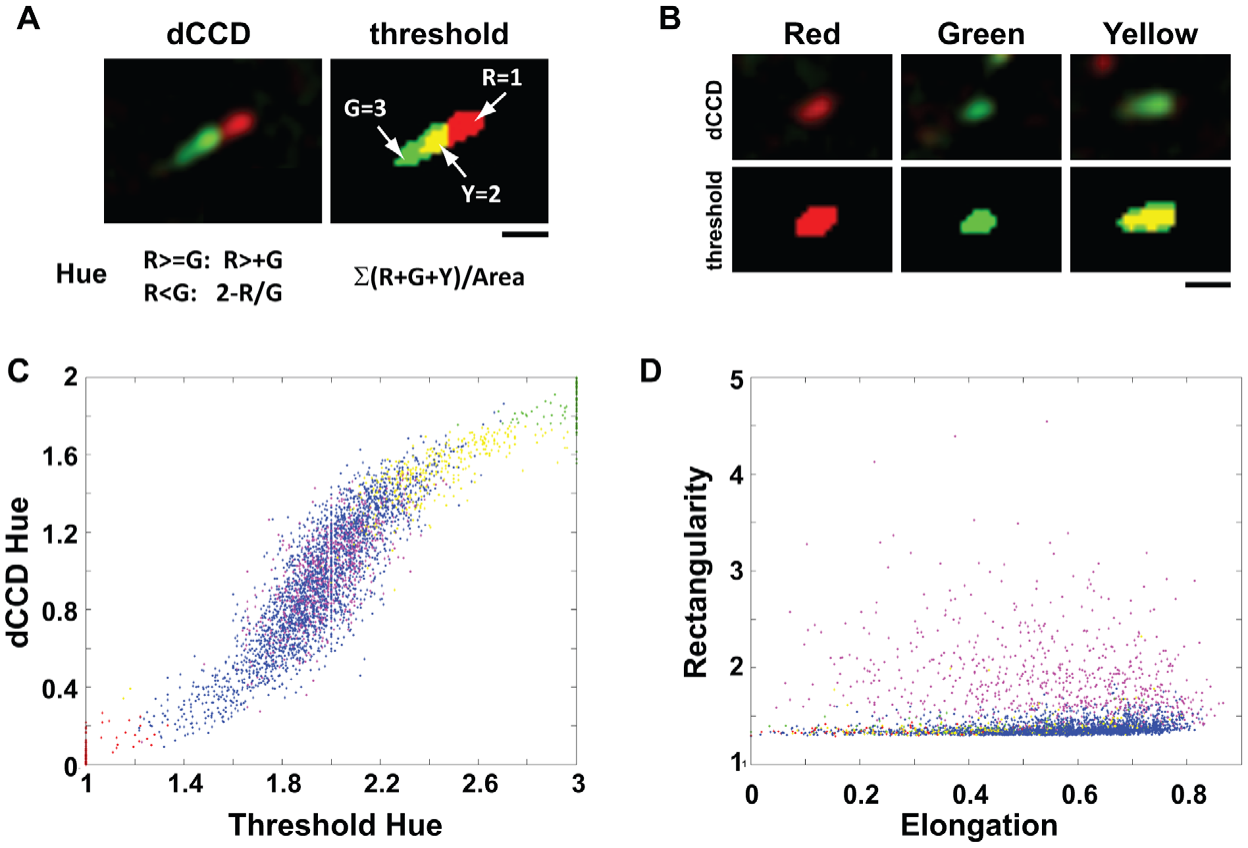
Color and morphological analysis of the dCCD method. A–B . Analysis of color representation in each identified end. A green-red end (**A**) found in the dCCD image is thresholded to three colors: red, green, and yellow. The dCCD hue is calculated using the individual color intensity from the dCCD image in RGB format. Each color is also given a number (1, 2 or 3) for calculating the threshold hue, as indicated by the formula. Examples of red, green and yellow ends are shown in (**B**). Note that the green-red end contains a red head, a yellow transitional zone, and a green tail, while the yellow end contains no red head. **C–D.** Color and shape properties of all ends identified in a COS cell. The dCCD hue of each color-coded end is plotted against the threshold hue (**C**), while the rectangularity (a/(s*l), a = Area, s = short axis length, l = long axis length) is plotted against the elongation ((l-s)/l) (**D**). Red, green, and yellow ends are represented by corresponding colored dots, while single and clustered green-red ends are represented by blue and magenta dots respectively.

To identify and segregate color-coded labels, the dCCD images were enhanced by global thresholding with an intensity cut-off based on 95% of the individual color intensity. A user-defined adjustment variable (0–20%) was used to further raise the threshold cut-off if necessary. The resulting enhanced dCCD images have three thresholded colors (green, red, and yellow) in a black background (Figure S2C). Specific erosion with an adjustable area variable (default = 6 pixels) was used to remove non-specific signals. Numerical values were next assigned to each color in the enhanced dCCD images: 1 for red, 2 for yellow, 3 for green. Based on these values, color-coded ends were separated from the background and identified as individual ends (Figure S2D). Each end was then analyzed for mean numerical value (threshold hue), absolute pixel number (total area), and percentage of total pixel number for each color (relative area). Based on color and morphological criteria (Figure 2), they were sorted into four color groups: green-red, red, green, and yellow (Figure S2E–I). The green-red ends were further divided into singles and clusters based on their shape and size (Figure S2E, F). Individual ends in clusters were further separated after analyzing their area and shape (details will be described elsewhere). Sorting and background correction were made based on hue and intensity of each end in dCCD images.

### Error Analysis

To quantify errors in identifying microtubule ends, we compared randomly selected regions of dCCD and raw images using circle or boundary overlays (Figure S3). Using an object counter plugin of Image J (NIH and McMaster Biophotonic Facility), we counted ends found in both images as overlapping ends (O). We also found ends appearing in dCCD images but not in raw images and counted them as false positives (P), while those present in raw images but not identified by the dCCD method as false negatives (N). False positive rates are then calculated as (1-O/(O+P)), and false negative rates as (1-O/(O+N)) (Table 1).

**Table 1:** Comparison of error rates in identification of microtubule ends in COS cells.

Comparison	False Positive	False Negative
dCCD vs GTD	3.7±0.7%	4.0±0.6%
plusTipTracker vs GTD	1.8±0.4%	10.3±0.9%

Microtubule ends identified by the dCCD method or the plusTipTracker program are compared with ground-truth data (GTD) based on human identification in raw images (as illustrated in Figure S3). False positive rates are calculated as (1-O/(O+P), while false negative rates as (1-O/(O+N)), where O is the number of ends found by the dCCD method and GTD, P is the number only found by the dCCD method, and N is for those missed by the dCCD method. Data expressed in mean±s.e were pooled from randomly selected regions (∼8% of the total imaging area per frame, with 2160 ends examined) of 10–11 frames from 3 COS cells.

**Figure 3:**
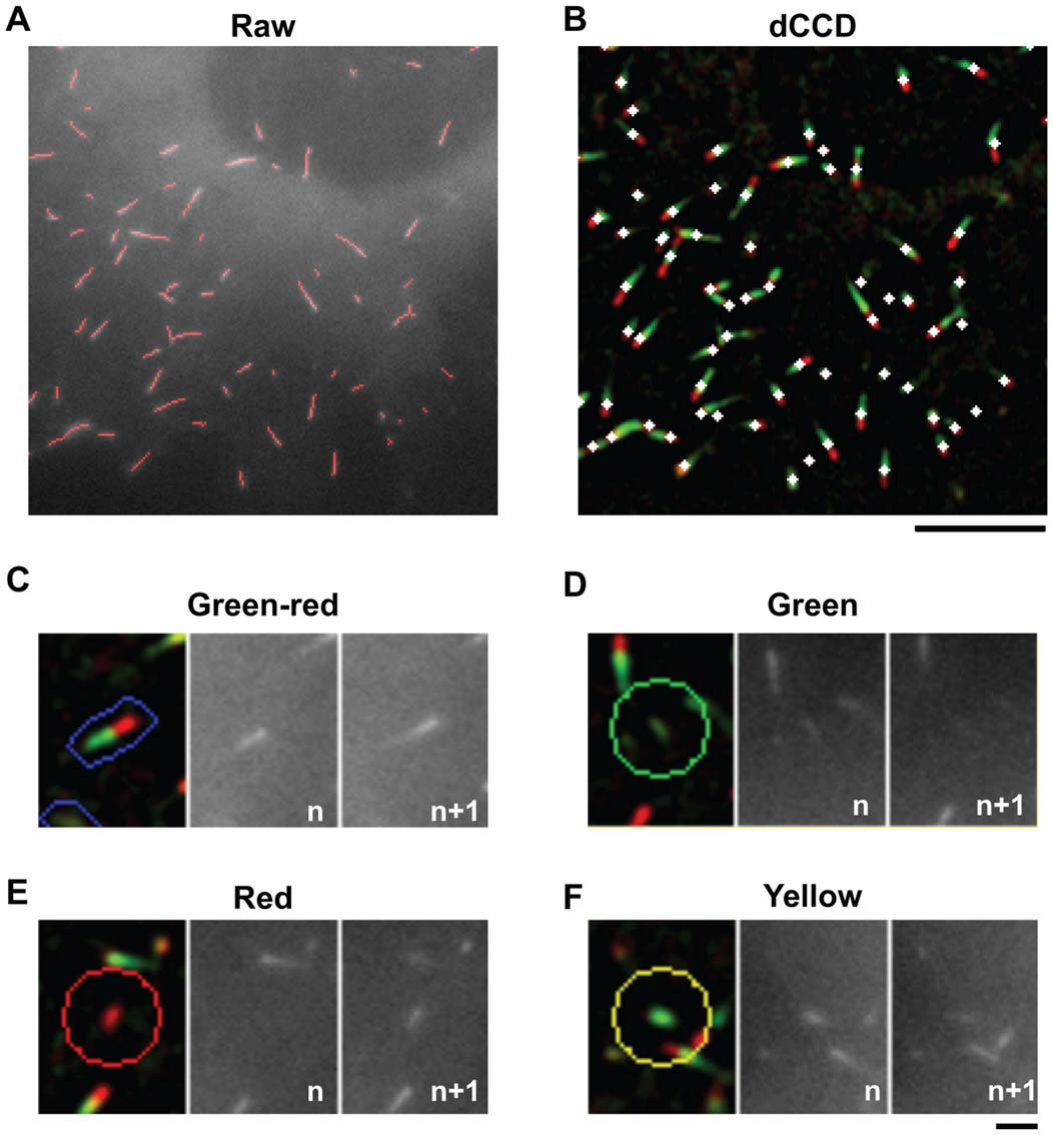
Evaluation of microtubule ends identified and segregated from dCCD images. A. Overlay of identified ends in skeletonized forms (red lines) on the raw fluorescent image in a subregion of a COS cell from Figure 1B. To match the frame in the raw image, identified ends were extracted from the green channel in the enhanced dCCD image (Figure S2C). Note nearly all visible EB3-GFP labels are marked. **B.** Centroids (white diamonds) of identified ends are overlaid onto color-coded ends in the dCCD image in the same region as in (a). Nearly all color-coded ends are marked. **C–F.** Validation of color codes assigned by the dCCD method. Colored ends segregated by the dCCD programs are represented by stretched boundaries (for green-red ends) or circles (for red, green, or yellow ends) surrounding the centroid. They are overlaid on top of dCCD images (left) and compared with the corresponding region in raw images (*n*th and *n+1*th frames). Scale bar: 1 µm.

To assess the fidelity of segregation, we overlaid identified ends shown in circles or boundaries on top of dCCD images (Figure 3C–F). Those ends sorted into wrong color groups were identified by visual evaluation of the overlay images. In addition, the same ends in the raw images (Figure 3C–F, two right panels) were analyzed to determine if color assignments actually matched their representation. For quantification, we calculated the accuracy rate by dividing the number of correctly sorted ends over the number of correctly identified ends in each color group (Table 2).

**Table 2:** Accuracy in segregation of color-coded microtubule ends.

Color Group	Accuracy
Green (214)	98.1±0.8%
Red (199)	98.5±1.0%
Yellow (253)	97.7±0.9%
Green-red (667)	99.4±0.3%

Segregation accuracy was analyzed in three COS cells as illustrated in Figure 3C–F, and calculated as described in Materials and Methods. Data were pooled from studies of randomly selected ends identified by the dCCD method (10–24 frames/cell) and expressed as mean±s.e. Numbers in parenthesis indicate the number of ends analyzed.

### Numerical Simulation

Based on a simple model, numerical simulations were done using 1000 randomly generated ends with normally distributed lengths and growth rates. The mean length used was 10±3 pixels. It is similar to that found in the sample cell and equivalent to 1 µm for a camera resolution of 0.1 µm/pixel. Growth rates were set between 2–20 µm/min.

### Statistical Analysis

To calculate identification and segregation errors, randomly selected regions or ends were analyzed in each frame. For history and fate analysis, randomly selected ends in each color group were analyzed in the −1 and +1 frame and scored based on their color representations. In both cases, data from 11–12 frames each of three cells were averaged and expressed as mean±s.e. Average growth rates and directional angles were calculated from each frame or multiple frames and expressed in mean±s.d.

## Results

### General Strategy of the dCCD Method to Visualize Dynamic Microtubule Ends

The dCCD method is based on the three possible events that can happen to a microtubule plus-end: growth, pause, and catastrophe for a growing microtubule, and shrinkage, pause, and rescue for a shrinking one (Figure 1A) [1]. Its use can be demonstrated in cells expressing EB3-GFP (Figure 1B). Fluorescent images of these labels are first processed using the difference of two Gaussian filters as recently described [13]. Two sequential images separated by an appropriate interval (for details, see discussion below) are then pseudocolored individually with different primary RGB colors (such as green for the *n*th frame and red for the *n+1*th frame). They are finally merged to generate a dCCD image containing labeled ends with different color combinations or codes (such as green-red, red, green, and yellow).

These color codes are the result of movement and appearance/disappearance of EB3-labels and provide a simple way to identify four of the six dynamics events illustrated in Figure 1A. For example, the majority of colored ends in the dCCD image appear green-red, with a red head and a green tail often connected by a yellow transitional zone (Figure 1B, solid rectangle). These are growing plus-ends with EB3-GFP labels associated with advancing microtubules, colored green in the *n*th frame and red in the *n+1*th frame. Two other populations appear to have a single color, either red or green, with varying intensities. Red (Figure 1B, solid circle) represents ends that have just regained EB3-GFP labels, likely being rescued after shrinkage and hence showing only in the *n+1*th frame. Green (Figure 1B, dashed circle) marks those that have lost EB3-GFP in the *n+1*th frame and likely indicates catastrophe, as +TIP proteins normally dissociate from depolymerizing ends and thus are only present in the *n*th frame [10]. Finally, a small number of ends appear yellow throughout the entire end, mostly greenish yellow (Figure 1B, dashed rectangles) without noticeable red heads. These are pausing ends with EB3-GFP present at the same location in both frames and hence overlapping of red and green in the dCCD image. Thus, this color-coding method provides a rapid way to visualize different dynamic microtubule ends in space.

### Identification and Segregation of Different Dynamic Microtubule Ends by Color and Morphological Analysis

The four-color groups found in the dCCD images can be identified and segregated by using object recognition algorithms based on color and morphological properties, such as hue, intensity, size, and shape. Red or green labels can be identified and separated from other color groups based on both dCCD and threshold hues, which are computed from the red and green intensity of each color-coded end in the dCCD image or the numerical value assigned in the thresholded image (Figure 2A–C). Yellow labels can be separated from green-reds based on the relative and absolute area of the red color within the object boundaries, because they do not have any distinguishable red head (Figure 2B). Green-red ends can be further separated into two subgroups, including singles (only one end) and clustered (two or more crossing ends) (Figure S2E–F), based on rectangularity (defined as the ratio of the area over the length multiple of the short and long axes) but not elongation (defined as the length ratio of the short and long axes difference over the long axis) (Figure 2D, blue vs. magenta).

These color and morphological criteria provide a reliable way to automatically identify and segregate labeled microtubule ends with reasonable accuracy. This can be demonstrated by visually inspecting the overlaps between the identified ends and those in the raw or dCCD images (Figure 3A–B) as well as by comparing dCCD images with the two raw images used to generate them (Figure 3C–F). To quantitatively assess identification errors, we developed a systematic way to randomly analyze all ends from each time point (or dCCD frame) and compare them to ground-truth data derived from human analysis (see Materials and Methods and Figure S3). Of 2160 microtubule ends from ∼30 dCCD images analyzed, the percentage of false positives is 3.7±0.7% and the percentage of false negatives is 4.0±0.6% (Table 1). For comparison, the same analysis was done with microtubule ends identified by the plusTipTracker program [13], [17] (Figure S3), and the errors are 1.8±0.4% and 10.3±0.9% respectively (Table 1).

In addition, we analyzed the segregation accuracy for each color group based on both dCCD and raw images (Figure 3C–F). Of randomly selected ends (n = 1333) in ∼30 frames, >97% are correctly segregated for each color group (Table 2), indicating that different color-coded ends can be reliably sorted by color and morphological analysis. On close inspection, most segregation errors came from rapidly growing ends in which the green and red representation of EB3-GFP labels from the same microtubule were disconnected and recognized by the program as two separate ends as described below. Therefore, the color coding scheme provides sufficient information for accurate identification and segregation of different dynamic ends.

### Validation of Dynamics at Color-coded Ends

**Figure 4:**
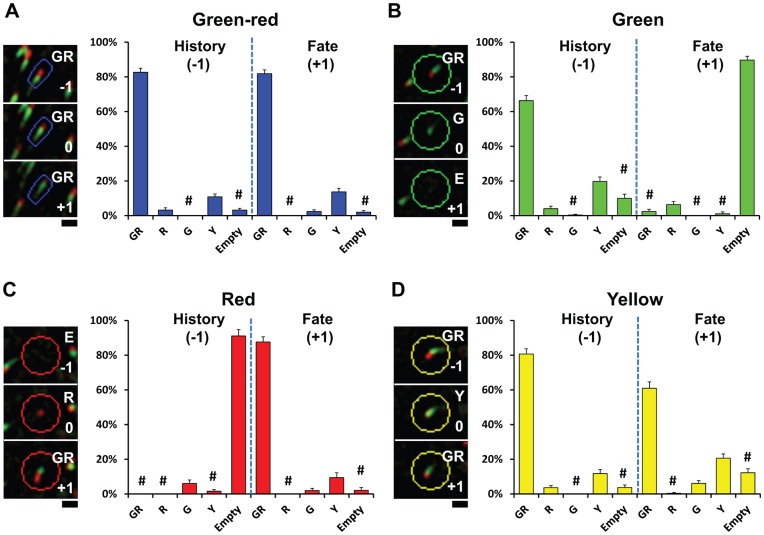
History and fate analysis of color-coded ends. Analysis of microtubule end behaviors for all four color groups. Color-coded ends identified in the 0 frame from each group ((**A**) for green-red, (**B**) for green, (**C**) for red, and (**D**) for yellow) are analyzed for their color codes in the −1 frame for history or in the +1 frame for fate. Left panels are sample images used for the analysis based on dCCD images, while right panels show quantification of behavior based on the percentage (mean±s.e.) of five possible events found. These events were tallied based on the following color codes: growth = green-red (GR), pause = yellow (Y), catastrophe = green (G), shrink = no label (Empty or E), and rescue = red (R). Events that do not match the predicted history or fate in Figure S4 are marked by #. Scale bar: 1 µm.

To verify that the color codes assigned by the dCCD method accurately represent the dynamic events proposed in the model, we next characterized the behaviors of each colored end (identified in the 0 frame) by examining its immediate history (from the −1 frame) and fate (from the +1 frame) in dCCD images (Figure 4). First, we examined green-red ends and found most of them (82.7±2.4%) came from growing microtubules in the −1 frame, corresponding well with their expected behavior (Figure 4A). Two small populations came from pausing (yellow, 11.9±1.6%) or rescued ends (red, 3.2±1.3%). In the +1 frame, the majority (81.9±2.2%) of green-reds continued to grow while 13.7±2.0% paused (yellow), whereas <3% lost EB3 labels and appeared green, a sign indicating catastrophe.

We also followed red ends (Figure 4C) and found that 91.1±3.7% of ends were from shrinking microtubules, lacking any EB3 label in the −1 frame. Approximately 6.1±2.0% came directly from green ends, indicating they were rescued immediately instead of shrinking after losing EB3-GFP labels. Following the rescue, 87.7±3.0% of red ends continued to grow in the +1 frame, consistent with the behavior of rescued microtubule ends. Interestingly, 9.4±2.8% of them became yellow while another 2.0±1.2% appeared green.

Green and yellow ends have different fates despite sharing a similar history, as both primarily emerge from growing microtubules (66.3±2.7% and 80.7±3.0% respectively). The majority (89.7±2.2%) of green ends do not regain any EB3-GFP labels (Figure 4B), implying they are truly undergoing catastrophe in the 0 frame. In contrast, only 6.0±1.6% of yellow ends undergo catastrophe, while 20.6±2.4% remain in the pausing phase, and 60.9±3.7% resume growth (Figure 4D), indicating they are similar but not identical to growing (green-red) ends.

Behaviors of all color codes correlate well with the predictions (Figure S4) of the corresponding dynamic events based on the color-coding model (Figure 1A). Of all ends analyzed, only 2.5±0.3% of total behaviors fall outside the predicted history and fate (Figure 4A–D, columns labeled by #), indicating the accuracy of the dCCD method. Thus, the color codes can be used to identify different dynamic events and will be used to refer them in the rest of the study.

### Quantitative Analysis of Microtubule Dynamics by the dCCD Method

**Figure 5:**
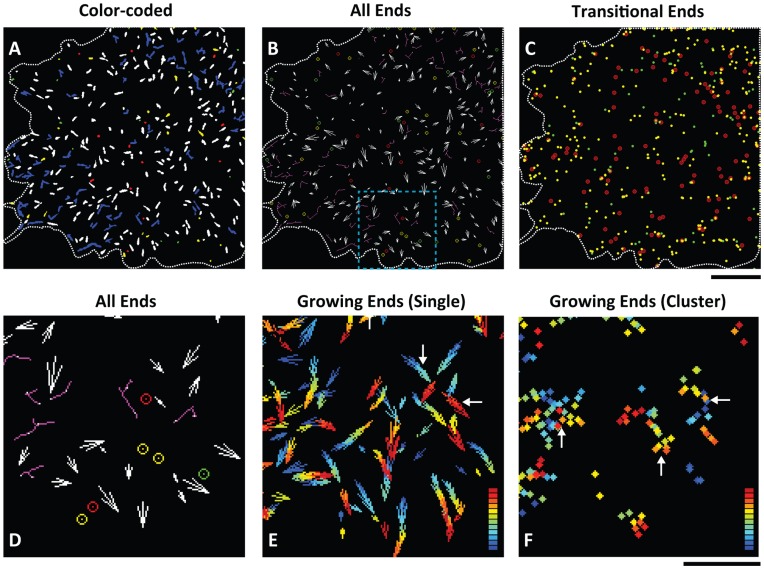
Quantitative analysis of microtubule dynamics in a COS cell. A. Spatial distribution of all dynamic microtubule ends identified at a single time point from a COS cell using the dCCD method (Figure S2). All ends are shown by their size and shape. The associated colors represent the dynamics groups each end belongs to: white for single growing ends, blue for clustered growing ends, red for rescued ends, green for those in catastrophe, and yellow for pausing ends. **B.** Visualization of microtubule ends in space by their dynamic properties in a single frame of the COS cell in (A). The centroids of three transitional events (red, green, and yellow) are shown in circles of corresponding colors. Single growing ends are shown in white growth vectors to indicate their growth direction (arrow orientation) and rate (arrow size), while clustered growing ends are shown in magenta skeletons with identified ends labeled by white dots. The dotted blue box indicates the region enlarged in (**D–E**). **C.** Centroids of microtubule ends undergoing rescue (red circles), catastrophe (green dots), and pause (yellow dots) in the same COS cell are pooled from 12 sequential frames and merged together to show their spatial distribution. **D.** High magnification picture from the boxed region found in (**B**). **E.** Visualization of single growing ends in time and space. Single growing ends identified in the boxed region in (B) are pooled together from 12 frames (1 min) and shown by their growth vectors. The associated color represents their frame sequence in reference to a color-based time map, in which blue indicates the first frame and red represents the 12th frame. Many ends aligned perfectly along their growth trajectory toward the cell periphery, revealing their continuous polymerization in the cell. Newly growing ends (red ends, horizontal arrow) are distinguishable from old ones (blue ends, vertical arrow). **F.** Visualization of clustered growing ends by their centroids in the boxed region in (**B**). Centroids from 12 frames (1 min) are shown in color based on a colored time map. Note many centroids are aggregated in patches (arrows). Scale bars: 10 µm for (**A–C**), 5 µm for (**D–F**).

The color codes generated by the dCCD method capture dynamic events at each labeled plus-end, making it feasible to quantitatively investigate microtubule dynamics in space, which is illustrated here by the analysis of COS cells (Figure 5). First, the dCCD method reveals the spatial distribution of all four dynamic ends at any given time (Figure 5A–B), and calculates the proportion of each event in the entire population of labeled ends. Here in the sample cell, nearly 87% of all identified ends at a single time point (n = 416) are growing (green-red), while the remaining ends are in rescue, catastrophe and pause (3.1% red, 3.6% green, and 6.3% yellow respectively). In addition, the variation of each frame from the total mean is within two standard deviations (Figure S5), indicating that the dCCD method provides consistent population-wide statistics.

Second, the method is extremely useful in analyzing rare transitional events in space (rescue, catastrophe and pause) [1]. They can be visualized at each time point (Figure 5A–B) or combined from multiple frames (Figure 5C). In the example shown here, all three events are found throughout the cell when combined from 12 frames within a 1-min time period (Figure 5C). They are often located in the cell periphery, consistent with past observations based on individually tracked microtubules [18], [19]. In addition, rescue occurs more interiorly than pause and catastrophe, consistent with microtubules being rescued after shrinking rearwards for a distance. In addition, the ratio of rescue over catastrophe identified in every two dCCD frames (10-sec) is 1.02±0.06, indicating a balanced transition between growth and shrinkage.

**Figure 6:**
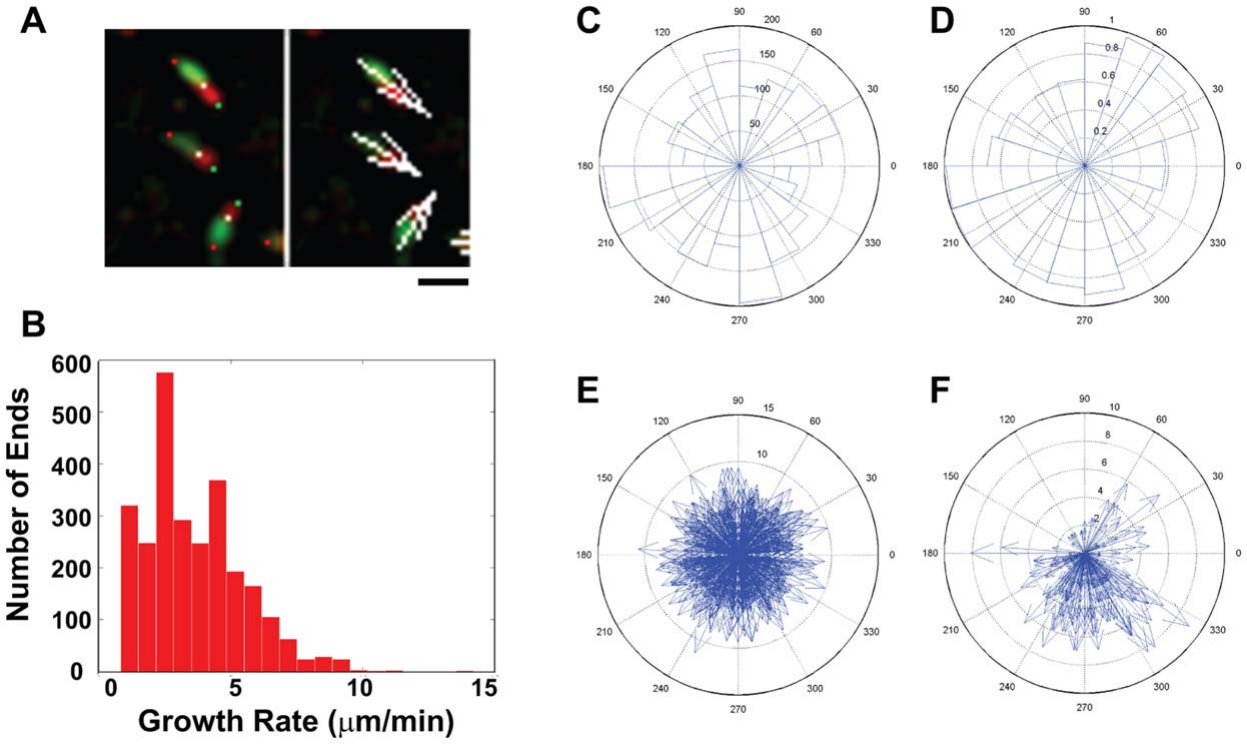
Analysis of microtubule growth rate and direction derived from single growing ends. A. Analysis of microtubule growth rate and direction in green-red ends. Rates are calculated as the distance between the front (green dots) and the rear (white dots) of red head, while directions are calculated from direction vectors connecting the rear of green tails (red dots) to the front of red heads (green dots). Growth vectors (shown by white arrows in left panels) are drawn from the back of green tails using normalized direction vectors scaled up to the quadruple of calculated rates. Scale bar: 1 µm. **B.** Histogram of growth rates calculated for all single green-red ends identified in the COS cell. **C–D.** Polar histogram plots of growth directions calculated for all single green-red ends from 12 dCCD frames of the COS cell in Figure S2A (**C**) or derived by the plusTipTracker program from the raw images of the same cell (**D**). **E–F.** Polar plots of growth rates against growth directions of single green-red ends from 12 dCCD images of the entire COS cell (E) or the boxed region (F) shown in Figure 5B. The numbers between 60° and 90° indicate the actual number of ends (**C**), the normalized end number ratio (**D**), or the growth rate in µm/min (**E–F**).

Third, growth direction and rate of single growing microtubule ends can be obtained from dCCD images (Figure 6). To do so, we first use morphological operations to identify the front of red heads in green-red ends (Figure 6A, green dots), and the rear of green tails (Figure 6A, red dots). Growth direction represented by the angle (0°–360°) is calculated based on direction vectors pointing from the rear of green tails to the front of red heads (Figure 6A, right panel). In the COS cell analyzed, the average growth angle is 184°±105° (single frame) or 176°±101° (multiple frames) for the entire cell, as expected for non-polarized microtubule assembly in a stationary cell. It is similar to that calculated by the plusTipTracker program [13] (Figure 6C–D), but different from that for ends found in the bottom region of the COS cell, indicating the accuracy of the method (Figure 6E–F). In addition, we identified the rear of red heads at the interface between red and yellow/green regions (Figure 6A, white dots). Growth rate is then calculated from the distance between the front and the rear of red heads. The rate average computed either from individual frames (3.7±1.9 µm/min) or combined from 12 frames (3.6±1.7 µm/min) (Figure 6B), is similar to that (3.7±1.5 µm/min) obtained using the plusTipTracker program [13].

Furthermore, growth vectors constructed at each end from growth direction and/or rate (Figure 6A) can be visualized in the entire cell (Figure 5B, D, E). When pooled together from multiple frames and labeled by time-mapped color, linear arrays of these growth vectors representing continuous polymerization can be visualized (Figure 5E, Figure S6A). Their spatiotemporal relationship can be detected, with more recent assembly shown in warm colors and those assembled earlier in cold colors (Figure 5E, horizontal vs. vertical arrow). The spatiotemporal distribution of crossing or touching ends can be also analyzed similarly based on their centroids. Not only do they associate with each other in individual frames (Figure 5B, 5D, magenta lines with white dots), but also remain clustered to form patches in the same locations over many frames (Figure 5F, arrows). These patches often located in the cell periphery (Figure S6B), suggesting the presence of subregions that promote interactions between growing ends.

### Analysis of Optimal Intervals Used for the dCCD Method

**Figure 7:**
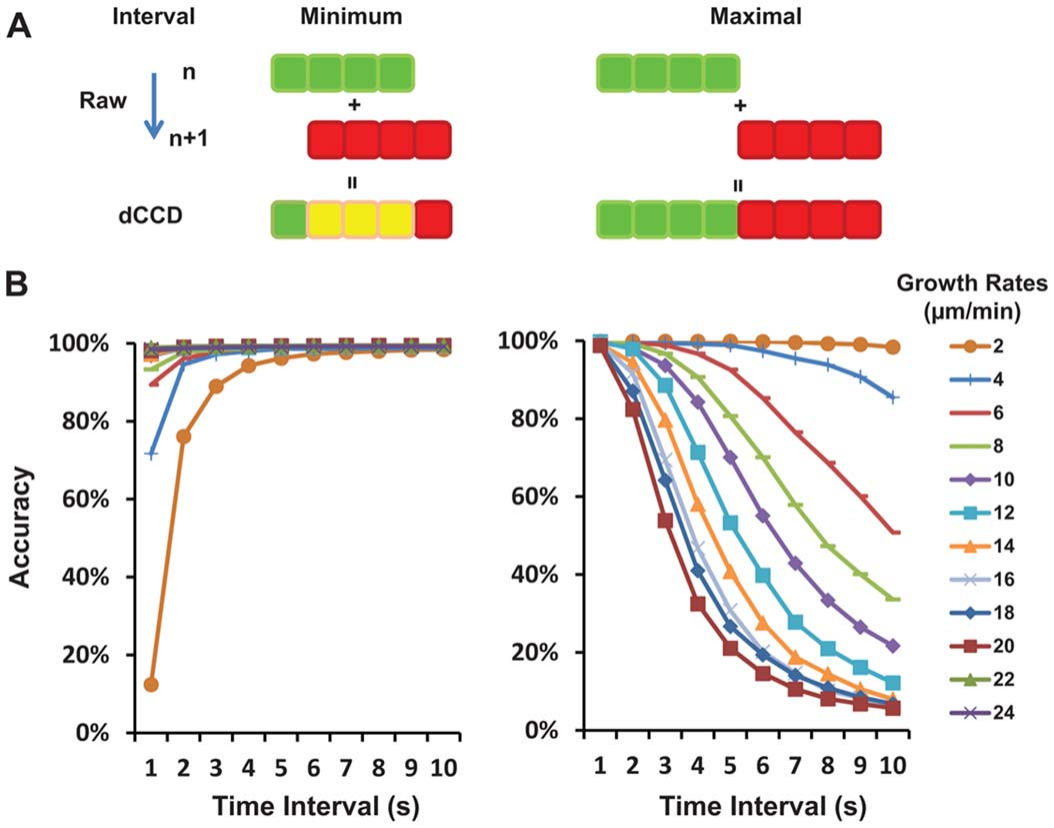
Analysis to identify minimum and maximal intervals for the dCCD method. A. A simple model to illustrate the limits of image intervals used for the dCCD method. Colored EB3-GFP labels are represented by linear arrays of colored squares equivalent to the pixels in digital images. The minimum interval (left) is defined as the time needed to detect the movement of the array from the *n*th frame (shown in green) to the *n+1*th frame (in red), leading to a new array with at least one red square followed by yellow and green squares in the merged dCCD images. The maximal interval (right) is defined as time allowed for the movement of one array length (4 pixels shown here) in the *n+1*th frame. **B.** Relationship of accuracy and time interval based on the numerical simulation using the criteria for minimum (left) and maximum (right) intervals defined in (**A**). For the minimum interval, accuracy is calculated as the number of ends that have moved at least one pixel length over 1000 total ends used in the simulation, whereas for the maximum interval, accuracy is calculated as the number of ends moved for less than the label length over 1000 simulated ends. Note the opposite relationship between the accuracy and the minimum or maximum interval. For fast growing microtubules (>14 µm/min), intervals >1 sec provide 98% accuracy for the minimum interval and <1 sec intervals give the same accuracy for the maximal interval. For slow growing ends found in the COS cell (4 µm/min), intervals between 3 sec and 7 sec can provide 95% accuracy.

While the dCCD method worked well with our EB3-GFP images, we wished to know the potential constraints of the method and to optimize image acquisition parameters. Because the method is based on the appearance/disappearance of the label as well as the spatial relationship of the same label in two sequential images, accurate representation of the four different dynamic groups depends on the time interval between two fluorescence images, the growth rate at microtubule ends (or moving rate of EB3-GFP labels), and the length of the labels. To find the optimal conditions, we first considered a simple model (Figure 7A). For a label with a fixed length (L pixels) and a fixed moving rate (R pixels/sec), the maximal interval that can be used is 2*L/R, so the label will not be separated into single green or single red. Conversely, the minimum interval needed is 1/R so the smallest movement of the label can be detected and the true growing end will not be sorted into the pause or yellow group. For a 1-µm long label moving at a rate of 10 µm/min and imaged with a camera resolution of 0.1 µm/pixel, the calculated minimum and the maximum interval are 0.5 sec and 5 sec respectively.

We next consider the entire microtubule population found in live cells, which usually have variable label length and growth rates. Thus, we used numerical simulation to determine the optimal intervals that can provide 95% accuracy for the dCCD method. Based on the above model with a normal distribution of growth rates and label lengths (1 µm), we found that 1-sec interval is optimal for any ends growing faster than 14 µm/min, while a range of intervals (from 5–10 sec to 1–2 sec) can be used for slower ends growing at 2–12 µm/min (Figure 7B). For the average growth rate (∼4 µm/min) found in the sample COS cell, the simulation suggests that 95% accuracy can be obtained using intervals between 3 sec and 7 sec, consistent with the analysis of our data (Table 1) and the condition used in our experiment.

## Discussion

Building upon recent studies using fluorescently tagged +TIPs to label microtubule plus ends [10], we have developed a computer vision strategy (Figure 1) to create snapshots of microtubule dynamics in space. Using image processing and object recognition algorithms (Figure 2), the dCCD method not only allows us to visualize microtubule dynamics in the entire cell, but also provides quantitative measurements (Figure 6). More importantly, the method is sensitive enough to obtain instant dynamics from only a pair of images, bypassing the need for frequent imaging used by tracking algorithms. Since the dynamics can be sampled in space at any time, this method will be useful to study microtubule regulation in fast moving and/or delicate structures such as growth cones.

The present study validates the approach of using color codes to visualize and quantify spatial regulation of microtubule dynamics. We have shown that color and morphological analyses work well to identify and segregate color-coded ends, with >95% accuracy when compared with ground-truth data (Figure 2–3, Table 1, 2). Using a simple model, we have identified optimal imaging parameters (intervals) that can be used to achieve 95% accuracy (Figure 7). In addition, measurements of growth rate and direction (Figure 6) are similar to those obtained by the plusTipTracker program [13].

Furthermore, we have demonstrated that color codes correlate well with four dynamic events based on history and fate analysis of microtubule ends (Figure 4). Since association of +TIPs with microtubule ends may vary in different conditions, such as pharmacological manipulations, these representations should be verified in future studies (see discussion below). Nonetheless, the dCCD method provides a way to identify different ends and facilitate their analysis. This will be especially useful for yellow ends, which were identified as pausing ends based on our behavioral analysis. However, they retain EB3-GFP (Figure 3,4) instead of losing +TIPs as previously reported for pausing microtubules [10], suggesting that they may have different EB3-GFP binding kinetics, perhaps as a result of different structural properties of these ends [20]. With the ability to detect changes in EB3-GFP association by color hue, the dCCD method will provide a tool to further investigate the underlying mechanism of this less understood event [18], [21], [22].

Finally, we have demonstrated the application of the dCCD method by analyzing microtubule dynamics in COS cells and obtained results consistent with past studies. For example, the rare transitional events are often associated with the cell periphery (Figure 5C), consistent with the previous life cycle study of microtubules [19]. In addition, spatial analysis reveals regions of the cells where growing microtubule ends tend to interact (Figure 5F), possibly regulated by other structures, such as actin and focal adhesions [23], [24], [25]. Spatial distribution, population statistics, and growth parameters thus provide rapid readouts of microtubule assembly, and can be used with different markers to reveal dynamic relationship between microtubules and other cellular structures.

While the dCCD method can be extended to other cell types and to other +TIP labels, some limitations should be noted. Due to the biochemical properties of +TIPs [10], the analysis does not provide measurements for all parameters traditionally used to describe microtubule dynamics [22]. For growing microtubules, the method calculates growth rate and direction as well as population frequency of pause or catastrophe. However, for shrinking microtubules, the method can reveal rescue but cannot provide pause frequency and depolymerization rate (Figure 1A). Also, color representation may be influenced by imaging conditions. For example, red or green ends can result from long interval between the two frames or from the +TIP labels entering or leaving the focal plane in a thick part of the cell. However, fine tuning of the experiment conditions, such as using shorter intervals (Figure 7) or optical sectioning, can improve the accuracy of color representation in these situations. Thus, we believe that the advantage of rapidly analyzing the entire population of +TIP-labeled microtubule ends outweighs these limitations, and when used appropriately, this method can complement the traditional measurements of individual microtubules.

In conclusion, we describe a simple color-coding method that is different from the commonly used tracking approach [12], [13], [14] and can detect microtubule dynamics from only a pair of plus-end labels. It reveals microtubule dynamics reliably at each labeled end and provides quantitative and spatial analysis of dynamic events at a given time. Therefore, the dCCD method provides a new tool to detect changes in microtubule dynamics and will be especially useful in studying microtubule dynamics during rapid structural remodeling.

## Supporting Information

Figure S1 **A flow chart to show the steps used in the dCCD method.** Five steps are involved in processing the raw image, generating and enhancing the dCCD images, identifying and segregating color-coded ends, and final analysis.
(PDF)

Figure S2 **Computer-assisted identification and segregation of color-coded microtubule ends by the dCCD method. A.** A grayscale raw fluorescent image of a COS cell expressing EB3-GFP. **B.** A dCCD image constructed by pseudocoloring processed raw images in green (*n*) and red (*n+1*, 5 sec). Plus ends are represented by comets in red, green, or combination. **C.** An enhanced dCCD image derived from (**B**) by thresholding and background reduction. **D.** A binary image of all microtubule ends identified by computer programs from (**C**). **E–I.** Microtubule ends identified in (**D**) are further segregated into four-color groups (green-red, red, green, and yellow). Note the green-red ends are further separated into single and clustered subgroups based on size and shape. Brown dashed lines indicate the cell boundary. Scale bar: 10 µm.
(PDF)

Figure S3 **Evaluation and validation of microtubule ends identified from dCCD images. A–D.** Identification analysis of randomly selected regions is shown by four examples (**A–D**). Ends identified by the dCCD method are shown by white dots and overlaid on dCCD images (left). The two raw images (n and n+1) used to generate the dCCD images are shown in the middle, which are used as references to determine identification error. Ends identified by the plusTipTrack programs are shown by blue diamonds and overlaid on top of the *n*th raw image (right). They are compared with ends identified by the dCCD method, which are shown in the same image by colored circles corresponding to green, red, or yellow ends or by colored boundaries for growing ends (blue for single and magenta for cluster). Black crosses to indicate the number ends found in the clusters.
(PDF)

Figure S4 **History (−1) and fate (+1) prediction of microtubule ends at different dynamic stages identified at frame 0. A.** Growing ends (green-red) are coming from microtubules that are growing (green-red), pausing (yellow) or being rescued (red). They can continue to grow (green-red), pause (yellow), or undergo catastrophe (green). **B.** Microtubules undergoing catastrophe (green) lose EB3-GFP when converting from growth (green-red), pause (yellow), or rescue (red) to shrinkage. They can stay in shrinkage, devoid of any EB3-GFP label (empty) or be rescued immediately (red). **C.** Rescued ends are mainly from shrinking microtubules (empty) or immediately from those undergoing catastrophe (green). They can continue to grow (green-red), pause (yellow) or convert to catastrophe (green). **D.** Like those in catastrophe or growth, pausing ends are from microtubules in growth (green-red), pause (yellow), or rescue (red), but their fate is similar to growing microtubules, ranging from continuing to grow (green-red), pausing (yellow), to undergoing catastrophe (green).
(PDF)

Figure S5 **Low variation in population analysis of color-coded ends.** Analysis of the variation in the distribution of four dynamic events in multiple frames or time points. The difference between the distribution of each frame and the mean of all frames is expressed as the number of standard deviation from the mean and plotted as a function of frames for each cell. Note that the differences for the four events shown by colored labels are mostly within two standard deviations for all three COS cells analyzed.
(PDF)

Figure S6 **Visualizing growing microtubule ends in time and space** **of the entire cell. A.** Single growing microtubule ends identified in the COS cell (Figure S2) from 12 frames (1 min) are shown together in arrows for their growth direction as well as in colors representing their sequence in time. Note many ends are linked together, revealing their continuous polymerization track in the cell. **B.** Centroids representing clustered growing microtubule ends identified in the COS cell (Figure S2) from 12 frames (1 min) are shown in colors representing their time sequence. Note many centroids are aggregated especially in the cell periphery. In both cases, the color map corresponds to the frames where the ends were identified. Blue indicates the first frame, while red color represents the 12th frame. White dashed lines indicate the cell boundary. Scale bar: 10 µm.
(PDF)

## References

[1] Desai A, Mitchison TJ (1997) Microtubule polymerization dynamics. Annual review of cell and developmental biology 13: 83–117. 10.1146/annurev.cellbio.13.1.83 9442869

[2] Mitchison TJ (1988) Microtubule dynamics and kinetochore function in mitosis. Annual review of cell biology 4: 527–549. 10.1146/annurev.cb.04.110188.002523 3058165

[3] Dent EW, Gupton SL, Gertler FB (2010) The growth cone cytoskeleton in axon outgrowth and guidance. Cold Spring Harbor perspectives in biology 3. 10.1101/cshperspect.a001800 PMC3039926 21106647

[4] Kaverina I, Straube A (2011) Regulation of cell migration by dynamic microtubules. Seminars in cell & developmental biology 22: 968–974. 22001384 10.1016/j.semcdb.2011.09.017 PMC3256984

[5] Schulze E, Kirschner M (1988) New features of microtubule behaviour observed in vivo. Nature 334: 356–359. 3393227 10.1038/334356a0

[6] Sammak PJ, Borisy GG (1988) Direct observation of microtubule dynamics in living cells. Nature 332: 724–726. 3357537 10.1038/332724a0

[7] Rusan NM, Fagerstrom CJ, Yvon AM, Wadsworth P (2001) Cell cycle-dependent changes in microtubule dynamics in living cells expressing green fluorescent protein-alpha tubulin. Molecular biology of the cell 12: 971–980. 11294900 10.1091/mbc.12.4.971 PMC32280

[8] Gierke S, Kumar P, Wittmann T (2010) Analysis of microtubule polymerization dynamics in live cells. Methods in cell biology 97: 15–33. 20719263 10.1016/S0091-679X(10)97002-7 PMC3495240

[9] Waterman-Storer CM, Desai A, Bulinski JC, Salmon ED (1998) Fluorescent speckle microscopy, a method to visualize the dynamics of protein assemblies in living cells. Curr Biol 8: 1227–1230. 9811609 10.1016/s0960-9822(07)00515-5

[10] Akhmanova A, Steinmetz MO (2008) Tracking the ends: a dynamic protein network controls the fate of microtubule tips. Nature reviews Molecular cell biology 9: 309–322. 18322465 10.1038/nrm2369

[11] Galjart N (2010) Plus-end-tracking proteins and their interactions at microtubule ends. Current biology : CB 20: R528–537. 20620909 10.1016/j.cub.2010.05.022

[12] Salaycik KJ, Fagerstrom CJ, Murthy K, Tulu US, Wadsworth P (2005) Quantification of microtubule nucleation, growth and dynamics in wound-edge cells. Journal of cell science 118: 4113–4122. 16118246 10.1242/jcs.02531

[13] Matov A, Applegate K, Kumar P, Thoma C, Krek W, et al (2010) Analysis of microtubule dynamic instability using a plus-end growth marker. Nature methods 7: 761–768. 20729842 10.1038/nmeth.1493 PMC3032800

[14] Neukirchen D, Bradke F (2011) Cytoplasmic linker proteins regulate neuronal polarization through microtubule and growth cone dynamics. The Journal of neuroscience : the official journal of the Society for Neuroscience 31: 1528–1538. 21273437 10.1523/JNEUROSCI.3983-10.2011 PMC6623617

[15] Mimori-Kiyosue Y, Grigoriev I, Lansbergen G, Sasaki H, Matsui C, et al (2005) CLASP1 and CLASP2 bind to EB1 and regulate microtubule plus-end dynamics at the cell cortex. J Cell Biol 168: 141–153. 15631994 10.1083/jcb.200405094 PMC2171674

[16] Stepanova T, Slemmer J, Hoogenraad CC, Lansbergen G, Dortland B, et al (2003) Visualization of Microtubule Growth in Cultured Neurons via the Use of EB3-GFP (End-Binding Protein 3-Green Fluorescent Protein). J Neurosci 23: 2655–2664. 12684451 10.1523/JNEUROSCI.23-07-02655.2003 PMC6742099

[17] Applegate KT, Besson S, Matov A, Bagonis MH, Jaqaman K, et al (2011) plusTipTracker: Quantitative image analysis software for the measurement of microtubule dynamics. Journal of structural biology 176: 168–184. 21821130 10.1016/j.jsb.2011.07.009 PMC3298692

[18] Shelden E, Wadsworth P (1993) Observation and quantification of individual microtubule behavior in vivo: microtubule dynamics are cell-type specific. The Journal of cell biology 120: 935–945. 8432733 10.1083/jcb.120.4.935 PMC2200071

[19] Komarova YA, Vorobjev IA, Borisy GG (2002) Life cycle of MTs: persistent growth in the cell interior, asymmetric transition frequencies and effects of the cell boundary. Journal of cell science 115: 3527–3539. 12154083 10.1242/jcs.115.17.3527

[20] Howard J, Hyman AA (2003) Dynamics and mechanics of the microtubule plus end. Nature 422: 753–758. 12700769 10.1038/nature01600

[21] Grabham PW, Seale GE, Bennecib M, Goldberg DJ, Vallee RB (2007) Cytoplasmic dynein and LIS1 are required for microtubule advance during growth cone remodeling and fast axonal outgrowth. The Journal of neuroscience : the official journal of the Society for Neuroscience 27: 5823–5834. 17522326 10.1523/JNEUROSCI.1135-07.2007 PMC6672755

[22] Straube A (2011) How to measure microtubule dynamics? Methods in molecular biology 777: 1–14. 21773917 10.1007/978-1-61779-252-6_1

[23] Efimov A (2008) Schiefermeier N, Grigoriev I, Ohi R, Brown MC, et al (2008) Paxillin-dependent stimulation of microtubule catastrophes at focal adhesion sites. Journal of cell science 121: 196–204. 18187451 10.1242/jcs.012666 PMC3164837

[24] Small JV, Kaverina I (2003) Microtubules meet substrate adhesions to arrange cell polarity. Current opinion in cell biology 15: 40–47. 12517702 10.1016/s0955-0674(02)00008-x

[25] Hoogenraad CC, Koekkoek B, Akhmanova A, Krugers H, Dortland B, et al (2002) Erratum: Targeted mutation of Cyln2 in the Williams syndrome critical region links CLIP-115 haploinsufficiency to neurodevelopmental abnormalities in mice. Nat Genet 32: 331. 10.1038/ng954 12195424

