# Supplementary figures and images for: Visualization and Analysis of Microtubule Dynamics Using Dual Color-Coded Display of Plus-End Labels
Source: PLoS One. 2012 Nov 30;7(11):e50421. doi: 10.1371/journal.pone.0050421 (PMC3511552; doi:10.1371/journal.pone.0050421)

Figure S1

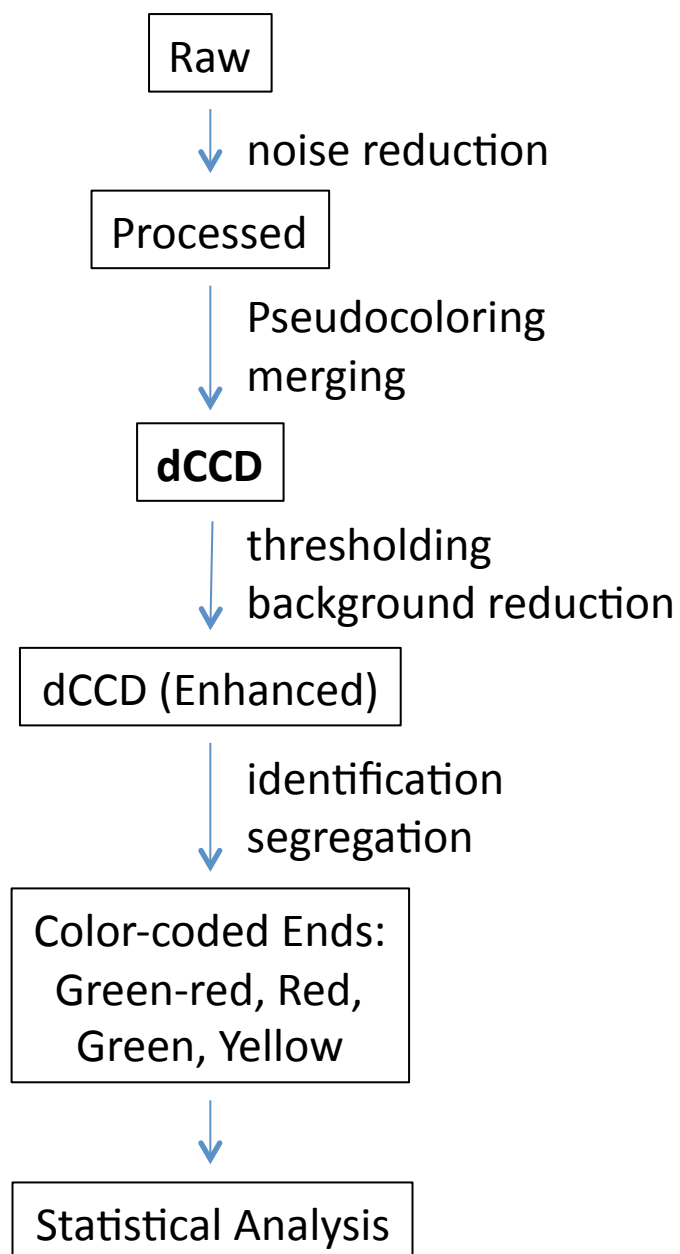

Supplement: Figure S1 — A flow chart to show the steps used in the dCCD method. Five steps are involved in processing the raw image, generating and enhancing the dCCD images, identifying and segregating color-coded ends, and final analysis. (PDF) [file pone.0050421.s001.pdf]

Figure S2

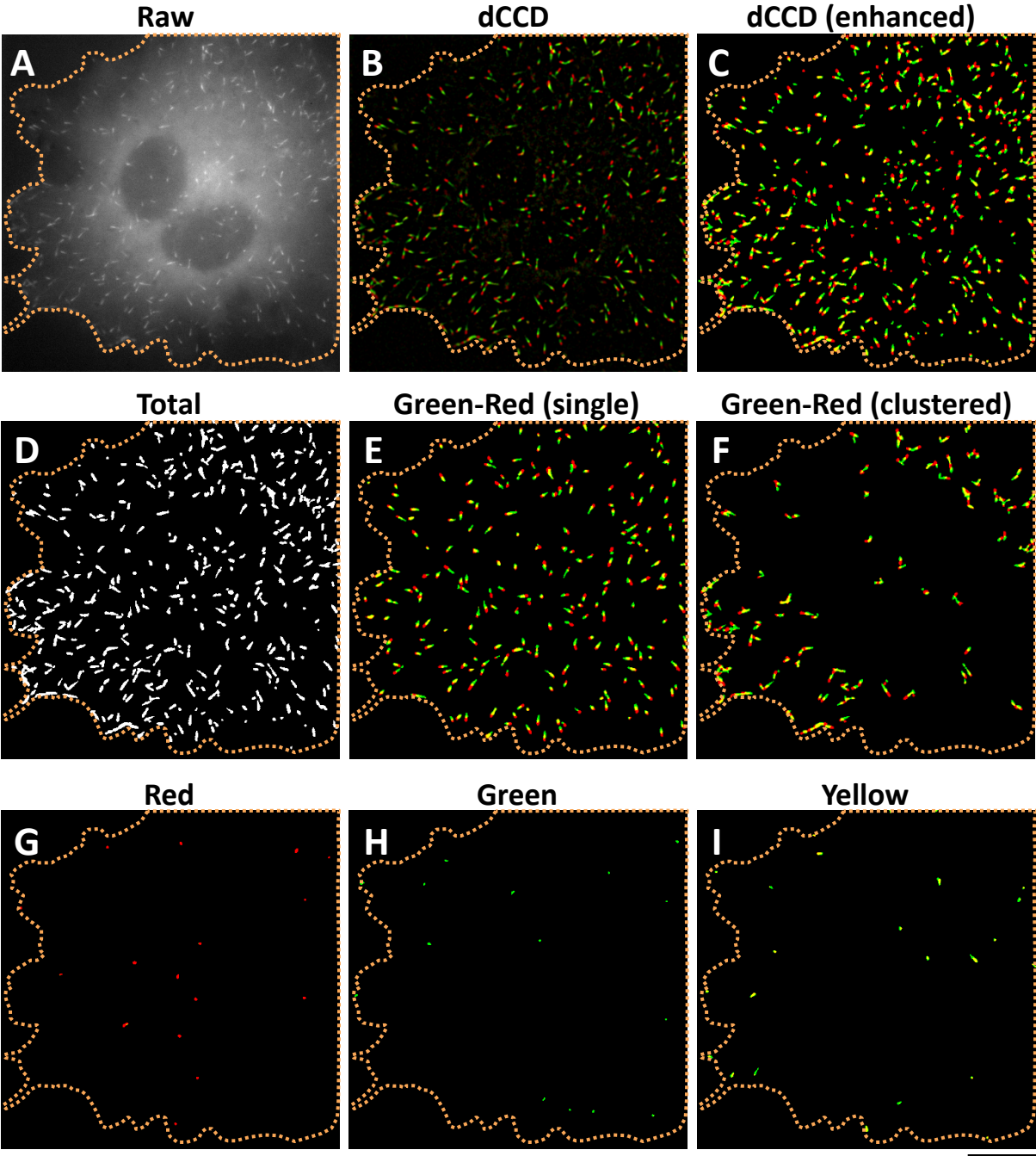

Supplement: Figure S2 — Computer-assisted identification and segregation of color-coded microtubule ends by the dCCD method. A. A grayscale raw fluorescent image of a COS cell expressing EB3-GFP. B. A dCCD image constructed by pseudocoloring processed raw images in green (n) and red (n+1, 5 sec). Plus ends are represented by comets in red, green, or combination. C. An enhanced dCCD image derived from (B) by thresholding and background reduction. D. A binary image of all microtubule ends identified by computer programs from (C). E–I. Microtubule ends identified in (D) are further segregated into four-color groups (green-red, red, green, and yellow). Note the green-red ends are further separated into single and clustered subgroups based on size and shape. Brown dashed lines indicate the cell boundary. Scale bar: 10 µm. (PDF) [file pone.0050421.s002.pdf]

Figure S3

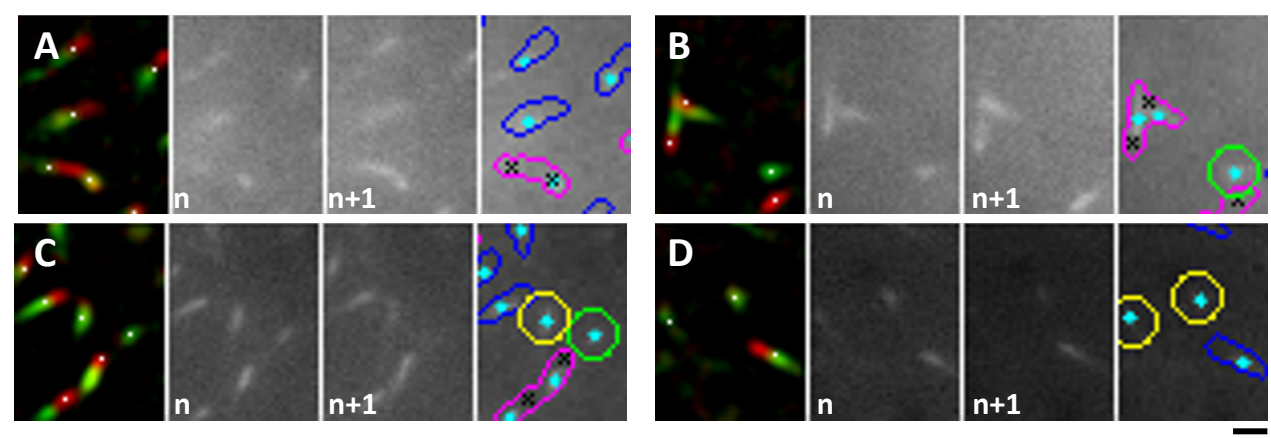

Supplement: Figure S3 — Evaluation and validation of microtubule ends identified from dCCD images. A–D. Identification analysis of randomly selected regions is shown by four examples (A–D). Ends identified by the dCCD method are shown by white dots and overlaid on dCCD images (left). The two raw images (n and n+1) used to generate the dCCD images are shown in the middle, which are used as references to determine identification error. Ends identified by the plusTipTrack programs are shown by blue diamonds and overlaid on top of the nth raw image (right). They are compared with ends identified by the dCCD method, which are shown in the same image by colored circles corresponding to green, red, or yellow ends or by colored boundaries for growing ends (blue for single and magenta for cluster). Black crosses to indicate the number ends found in the clusters. (PDF) [file pone.0050421.s003.pdf]

Figure S4

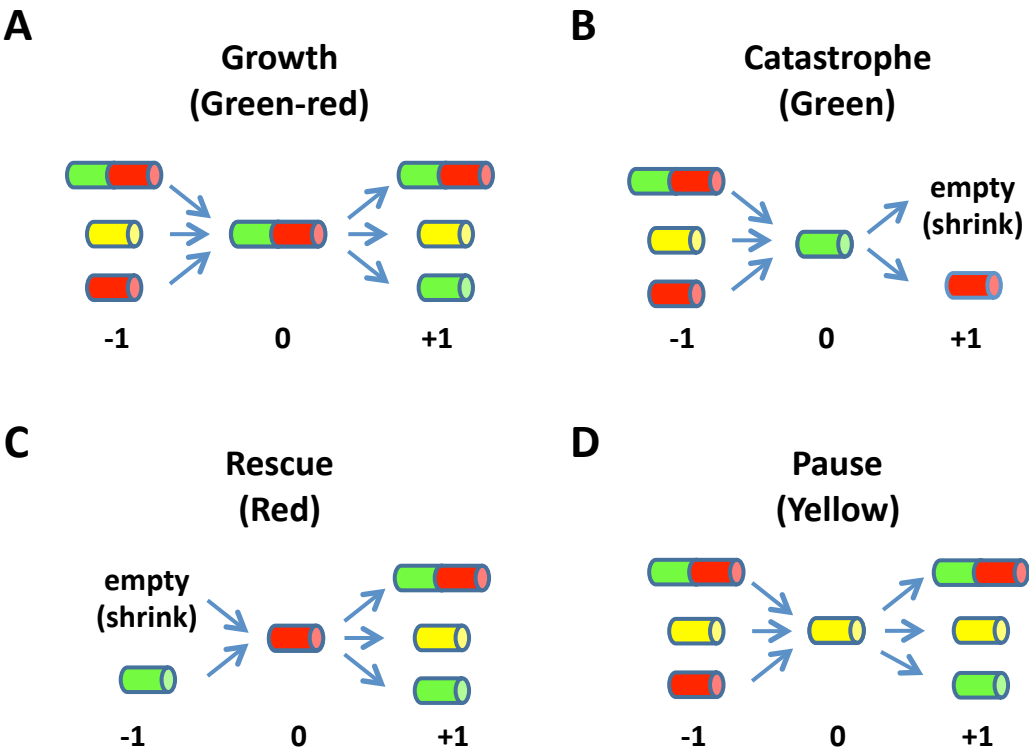

Supplement: Figure S4 — History (−1) and fate (+1) prediction of microtubule ends at different dynamic stages identified at frame 0. A. Growing ends (green-red) are coming from microtubules that are growing (green-red), pausing (yellow) or being rescued (red). They can continue to grow (green-red), pause (yellow), or undergo catastrophe (green). B. Microtubules undergoing catastrophe (green) lose EB3-GFP when converting from growth (green-red), pause (yellow), or rescue (red) to shrinkage. They can stay in shrinkage, devoid of any EB3-GFP label (empty) or be rescued immediately (red). C. Rescued ends are mainly from shrinking microtubules (empty) or immediately from those undergoing catastrophe (green). They can continue to grow (green-red), pause (yellow) or convert to catastrophe (green). D. Like those in catastrophe or growth, pausing ends are from microtubules in growth (green-red), pause (yellow), or rescue (red), but their fate is similar to growing microtubules, ranging from continuing to grow (green-red), pausing (yellow), to undergoing catastrophe (green). (PDF) [file pone.0050421.s004.pdf]

Figure S5

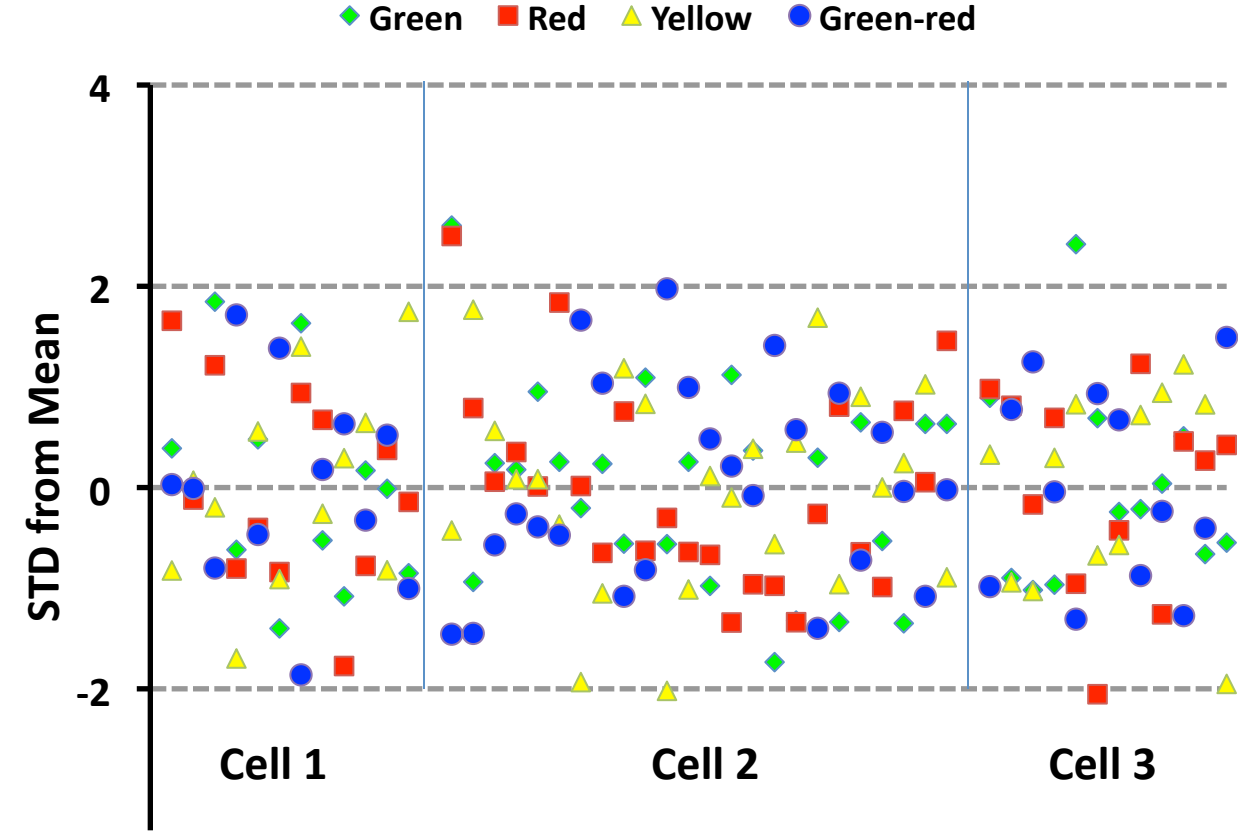

Supplement: Figure S5 — Low variation in population analysis of color-coded ends. Analysis of the variation in the distribution of four dynamic events in multiple frames or time points. The difference between the distribution of each frame and the mean of all frames is expressed as the number of standard deviation from the mean and plotted as a function of frames for each cell. Note that the differences for the four events shown by colored labels are mostly within two standard deviations for all three COS cells analyzed. (PDF) [file pone.0050421.s005.pdf]

Figure S6

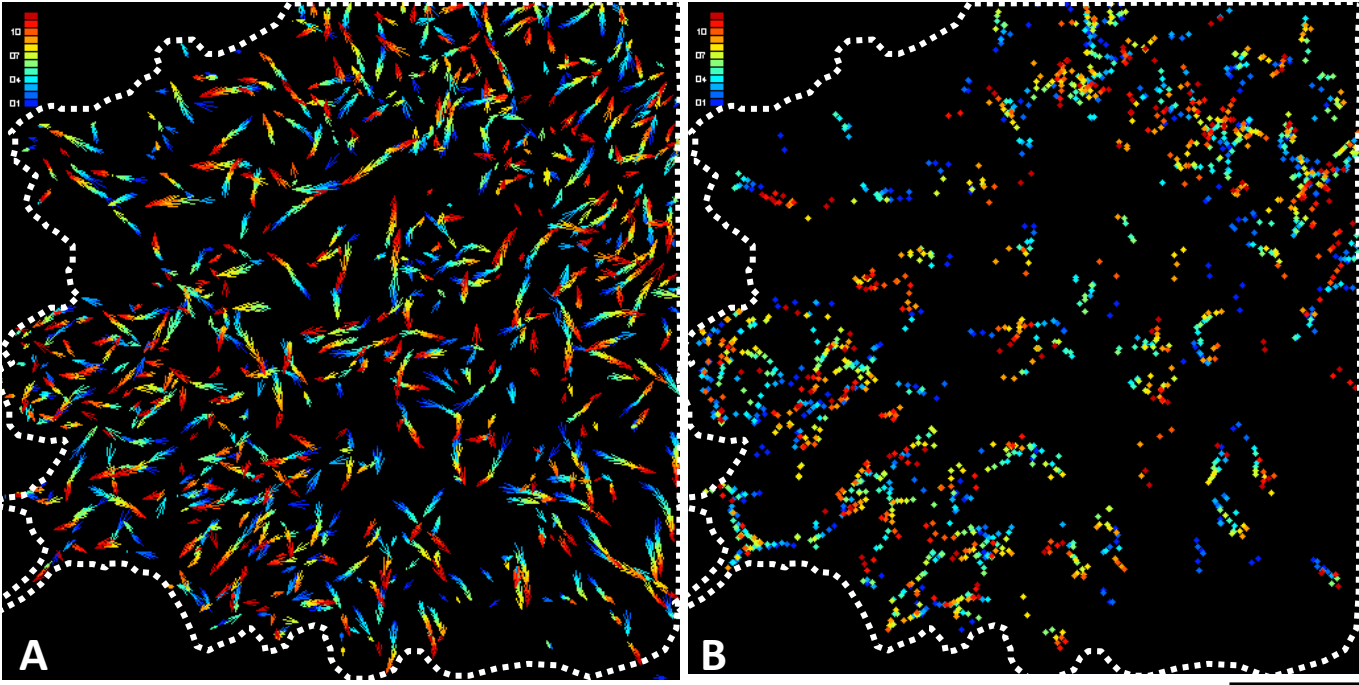

Supplement: Figure S6 — Visualizing growing microtubule ends in time and space of the entire cell. A. Single growing microtubule ends identified in the COS cell (Figure S2) from 12 frames (1 min) are shown together in arrows for their growth direction as well as in colors representing their sequence in time. Note many ends are linked together, revealing their continuous polymerization track in the cell. B. Centroids representing clustered growing microtubule ends identified in the COS cell (Figure S2) from 12 frames (1 min) are shown in colors representing their time sequence. Note many centroids are aggregated especially in the cell periphery. In both cases, the color map corresponds to the frames where the ends were identified. Blue indicates the first frame, while red color represents the 12th frame. White dashed lines indicate the cell boundary. Scale bar: 10 µm. (PDF) [file pone.0050421.s006.pdf]
